# Prolyl Isomerization-Mediated Conformational Changes Define ATR Subcellular Compartment-Specific Functions

**DOI:** 10.3389/fcell.2022.826576

**Published:** 2022-06-03

**Authors:** Himadri Biswas, Shu-Jun Zhao, Yetunde Makinwa, James S. Bassett, Phillip R. Musich, Jing-Yuan Liu, Yue Zou

**Affiliations:** ^1^ Department of Cancer Biology, University of Toledo College of Medicine and Life Sciences, Toledo, OH, United States; ^2^ Department of Medicine, University of Toledo College of Medicine and Life Sciences, Toledo, OH, United States; ^3^ Department of Bioengineering, University of Toledo College of Engineering, Toledo, OH, United States; ^4^ Department of Biomedical Sciences, Quillen College of Medicine, East Tennessee State University, Johnson City, TN, United States

**Keywords:** ATR, *cis/trans* prolyl isomerization, mass spectrometric protein footprinting, structure-function of ATR, mitochondrial ATR-tBid interaction, antiapoptosis, BH3-like domain, UV irradiation

## Abstract

ATR is a PI3K-like kinase protein, regulating checkpoint responses to DNA damage and replication stress. Apart from its checkpoint function in the nucleus, ATR actively engages in an antiapoptotic role at mitochondria following DNA damage. The different functions of ATR in the nucleus and cytoplasm are carried out by two prolyl isomeric forms of ATR: *trans*- and *cis*-ATR, respectively. The isomerization occurs at the Pin1 Ser428-Pro429 motif of ATR. Here, we investigated the structural basis of the subcellular location-specific functions of human ATR. Using a mass spectrometry-based footprinting approach, the surface accessibility of ATR lysine residues to sulfo-NHS-LC-biotin modification was monitored and compared between the *cis*- and the *trans*-isomers. We have identified two biotin-modified lysine residues, K459 and K469, within the BH3-like domain of *cis*-ATR that were not accessible in *trans*-ATR, indicating a conformational change around the BH3 domain between *cis-* and *trans*-ATR. The conformational alteration also involved the N-terminal domain and the middle HEAT domain. Moreover, experimental results from an array of complementary assays show that *cis*-ATR with the accessible BH3 domain was able to bind to tBid while *trans*-ATR could not. In addition, both *cis*- and *trans*-ATR can directly form homodimers *via* their C-terminal domains without ATRIP, while nuclear (*trans*-ATR) in the presence of ATRIP forms dimer–dimer complexes involving both N- and C-termini of ATR and ATRIP after UV. Structural characteristics around the Ser428-Pro429 motif and the BH3 domain region are also analyzed by molecular modeling and dynamics simulation. In support, *cis* conformation was found to be significantly more energetically favorable than *trans* at the Ser428-Pro429 bond in a 20-aa wild-type ATR peptide. Taken together, our results suggest that the isomerization-induced structural changes of ATR define both its subcellular location and compartment-specific functions and play an essential role in promoting cell survival and DNA damage responses.

## Introduction

The DNA damage checkpoint pathways check genome integrity and synchronize multiple cellular pathways to establish efficient repair of DNA damage (Zhou and Elledge, 2000; Zou and Elledge, 2003). The ATM (ataxia-telangiectasia mutated) and ATR (ATM and RAD3-related) kinases mediate checkpoint pathways and represent two vital DNA damage-dependent checkpoints. Both ATM and ATR are members of the phosphoinositide 3-kinase-like related kinase family. These pathways encompass series of DNA damage sensors, signal mediators and transducers, and downstream effector molecules (Zhou and Elledge, 2000; Zou and Elledge, 2003; Sancar et al., 2004; Wu et al., 2007). The ATR-dependent checkpoint pathway contributes to anticipating replication stress and responds mainly to DNA damage caused by genotoxins such as UV irradiation (Shiloh, 2003; Zou and Elledge, 2003; Abraham, 2004; Sancar et al., 2004). After replication stress and generation of single-stranded DNA (ssDNA), RPA (replication protein A) coats the ssDNA and recruits the ATR–ATRIP complex *via* ATRIP (ATR-interacting protein). ATRIP is the nuclear partner of ATR and recruits bound ATR to the DNA damage site, where ATR is autophosphorylated at its T1989 residue (Cortez et al., 2001). This phosphorylated residue serves as a docking site for TopBP1 to substantially elevate the activation of ATR’s kinase activity (Burrows and Elledge, 2008; Mordes et al., 2008; Liu et al., 2011a). Activated ATR further triggers several key downstream proteins, including p53 and other checkpoint kinases such as Chk1, causing an S-phase cell cycle arrest for the repair of the DNA damage or, apoptosis, in case of severe damage (Cortez et al., 2001; Zou and Elledge, 2003; Sancar et al., 2004; Mordes and Cortez, 2008; Mordes et al., 2008; Ciccia and Elledge, 2010; Nam and Cortez, 2011; Saldivar et al., 2017; Ma et al., 2018).

ATR that functions in the cytoplasm was found to play an important antiapoptotic role directly at the mitochondria, independent of nuclear ATR and kinase activity (Hilton et al., 2015). Except for its synthesis, ATRIP is largely absent in the cytoplasm. Contrary to nuclear ATR that always remains in the *trans*-form in a complex with ATRIP, the cytoplasmic ATR, devoid of ATRIP, exists in two prolyl isomeric forms, the *cis*- and *trans-*form. The existence of both these forms depends on a single-peptide bond orientation in ATR by prolyl isomerization. In contrast, nuclear ATR always remains in the *trans*-form in a complex with ATRIP. Pin1 regulates the balance between the *cis* and *trans* cytoplasmic form of ATR and catalyzes this conversion of *cis*-ATR to *trans*-ATR by recognizing the phosphorylated Serine 428-Proline 429 residues (pS428-P429) in the N-terminal region of ATR (Hilton et al., 2015). Albeit Pin 1 activity favors *trans*-ATR formation when it is inactivated by DAPKI kinase, in response to DNA damage, it promotes *cis*-ATR accumulation at mitochondria as *cis*-ATR is more stable in cells. Contrary to its *trans*-ATR isoform, the *cis*-ATR has an exposed BH3-like domain allowing it to bind to the pro-apoptotic tBid protein at the mitochondria (Hilton et al., 2015; Musich et al., 2017; Makinwa et al., 2020a; Makinwa et al., 2020b). This binding prevents tBid from activating Bax–Bak polymerization, which is vital for the intrinsic apoptotic pathway. Here forth, *cis*-ATR executes an antiapoptotic function that protracts the cell survival long enough to repair its damaged DNA. However, this can be a double-edged sword that can play a role in carcinogenesis. The newly discovered BH3 domain, a hallmark of apoptotic proteins, in ATR defines *cis*-ATR’s role in the apoptosis pathway. However, the structural characteristics of *cis*- and *trans*-ATR and the related functions of ATR are to be characterized yet.

In this study, we determine the structural alterations of two prolyl isomeric forms of ATR, *trans*- and *cis*-ATR, using mass spectrometric protein footprinting and complementary methods. Our results demonstrate a significant conformational change at the region containing the BH3 domain between the *cis* and *trans isomeric forms of* ATR, resulting in activation or silencing of the BH3 domain as required for ATR–tBid interaction at mitochondria. In addition, we found that *cis*- and *trans-*ATR isomers form distinctly different dimers in the presence and absence of ATRIP. The *cis*-ATR forms a homodimer *via* its C-terminal domain in the cytoplasm, while the latter, in the nucleus, dimerizes *via* both C- and N-terminal domains involving ATRIP and is a heterodimer–homodimer. Our results unravel the role of ATR at mitochondria as a pro-survival protein, broadening our understanding of the cellular functions of ATR.

## Experimental Procedures

### Cell Culture, UV Irradiation, and Antibodies

The simian virus 40-transformed human embryonic kidney (HEK) 293T cells and human colorectal carcinoma (HCT)116 ATR^flox/−^ cells were maintained in DMEM (GIBCO/BRL) and McCoy’s 5A media supplemented with 10% FBS and 100 units of penicillin and streptomycin/ml. UV treatments were at 40 J/m^2^ with a 2-h recovery. ATR, p-ATR (T1989), p-53(S15), p53, and tBid antibodies were purchased from Cell Signaling.

### Protein Purification

N-terminal Flag-tagged ATR recombinant protein was isolated from HEK293T cells 48 h after transfection with pcDNA3-Flag-ATR (WT), S428A, or P429A plasmid. The cells were lysed in ice-cold lysis buffer [50 mM HEPES, pH 7.4/150 mM NaCl/1 mM EDTA/10% glycerol/1% Triton-X100, and protease inhibitors (Thermo Fischer)] for 30 min. The cell lysates were centrifuged for 10 min at 20,000 × g. The supernatants from the spun lysates were incubated at 4°C overnight with magnetic anti-FLAG M2 affinity beads (Sigma) that had been pre-equilibrated in buffer containing 50 mM HEPES, pH 7.4/150 mM NaCl. The beads were then washed once with 1 M NaCl containing 50 mM HEPES, pH 7.4 buffer and thrice with 150 mM NaCl containing 50 mM HEPES, pH 7.4. The ATR bound to beads was either used directly for pull-down experiments or eluted with 50 mM HEPES, pH 7.4 buffer containing 200 μg/ml FLAG peptide (Sigma).

### Biotin Modification and In-Gel Proteolysis

Purified ATR and mutants were modified with various molar concentrations of sulfo-NHS-LC-biotin. Typically, ATR (2 μM) was modified by adding sulfo-NHS-LC-biotin (2 mM of final concentration) for 30 min incubation at room temperature. Modifications were quenched by the addition of 10 mM lysine, followed by purification of ATR by SDS-PAGE. The subunit bands on the gel were visualized by Coomassie blue staining, excised from the gel, and destained with water. SDS was removed by washing the gel pieces with ammonium bicarbonate, dehydrated with 100% acetonitrile, and vacuum desiccated. In-gel digestion with trypsin was performed using a ProGest robot (DigiLab). After trypsin digestion, the gel slices were washed with 25 mM ammonium bicarbonate followed by acetonitrile. Then, the gel pieces were reduced with 10 mM dithiothreitol at 60°C followed by alkylation with 50 mM iodoacetamide at RT. Thereafter, protein bands were digested overnight at 37°C with sequencing-grade trypsin (Promega). Finally, the peptide extract was dried and prepared for either MALDI-TOF or nanoLC MS/MS.

### MALDI-TOF

Peptide extracts were resuspended in 50% acetonitrile. One microliter of each peptide solution was spotted onto a Scout MALDI 384 target (Bruker Daltonics) and air-dried; 1.0 μL of α-cyano-hydroxycinnamic acid (10 mg∕mL) in 70:30 acetonitrile:0.1% trifluoroacetic acid was added to it. MALDI-TOF spectra were recorded by using a MALDI-TOF Biflex IV mass spectrometer (Bruker Daltonics) in positive ionization mode. Spectra of proteins and peptides were acquired in reflection mode (90–180 laser shots) in the m/z range from 500 to 3,500.

### Nano LC-MS/MS

Tryptic peptides were resuspended in 0.1% formic acid before loading onto a trapping column and eluted over a 75 µm analytical column at 350 nL/min; both columns were packed with Luna C18 resin (Phenomenex) and eluted over 40 min with a 3–40% acetonitrile gradient. The sample was analyzed by nano LC-MS/MS using a Waters NanoAcquity HPLC system interfaced to a ThermoFisher Fusion Lumos mass spectrometer. The mass spectrometer was operated in data-dependent mode, with the Orbitrap operating at 60,000 FWHM and 15,000 FWHM for MS and MS/MS, respectively. The instrument was run with a 3-s cycle for MS and MS/MS.

### Cytoplasmic and Nuclear Protein Extraction

To prepare samples for immunoprecipitation, subcellular fractionation was executed using cell lysis buffer [10 mM HEPES, pH 7.9, 10 mM KCl, 3 mM CaCl_2_, 1.5 mM MgCl_2_, 0.34 M sucrose, 10% glycerol, 0.1% Triton X-100, plus 1x protease, and phosphatase inhibitors (Thermo Fischer)] and a nuclear lysis buffer (50 mM Tris–HCl, pH 7.9, 140 mM NaCl, 3 mM CaCl_2_, 1 mM EDTA, 1% NP-40, 10% glycerol, plus 1x protease, and phosphatase inhibitors). Briefly, 10 volumes of cell lysis buffer were added to one volume of packed cells. After resuspension and incubation on ice for 10 min, the cytoplasm was separated from nuclei at 500 × g for 7 min at 4°C. Isolated nuclei were washed twice with 500 μL of the nuclear wash buffer (cell lysis buffer containing 30 mM DTT) and collected by centrifugation. Collected nuclear pellets were suspended in ice-cold nuclear lysis buffer, and nuclei were lysed with rotation for 40 min at 4°C. The nuclear lysate was clarified by centrifugation at 20,000 × g for 10 min at 4°C. Lysates were mixed with SDS-loading buffer to have the final composition of 450 mM Tris–HCl, pH 8.45, 12% glycerol, 4% SDS, 0.0075% bromophenol blue, and 100 mM DTT before heating at 95°C for 5 min and protein analysis by gradient (3–8%), Tris–acetate (TA) SDS-PAGE, and Western blotting (WB). ATR isomerization was always assayed by 3–8% TA SDS-PAGE (NuPAGE, Invitrogen) and analyzed by WB.

### Immunoprecipitation Assays

To assess tBid binding to ATR, the HCT116 ATR^flox/−^ cells were transfected with a plasmid construct of FLAG-ATR (wt), FLAG-ATR (S428A), or FLAG-ATR (P429A) and UV irradiated at 40 J/m^2^ followed by a 2-h recovery before the cytoplasmic fraction (including mitochondria) were collected. FLAG-beads (Sigma) were added for pull-down overnight. Immunoprecipitated (IP) FLAG-beads were washed three times in co-immunoprecipitation (Co-IP) wash buffer (50 mM Tris–HCl, pH 7.6, 140 mM NaCl, 1 mM EDTA, 10% glycerol, and 0.2% Tween-20). Purified tBid was added for a 2 h incubation at 4°C. The FLAG-beads were washed thrice using Co-IP wash buffer, followed by suspending in 1× SDS loading buffer and boiled at 95°C for 5 min before assaying the ATR isomerization status by 3–8% TA SDS-PAGE and analyzed by WB.

### Duolink *In Situ* Proximity Ligation Assays

The Duolink protein–protein interaction assay was performed according to the manufacturer’s instructions (Sigma DUO 92101). Images were captured using a Zeiss fluorescence microscope 40× objective lens. To determine ATR–Bid interactions among various ATR mutants by PLA, the HCT116 ATR^flox/−^ cells were transfected with a plasmid construct of FLAG-ATR (wt), FLAG-ATR (S428A), or FLAG-ATR (P429A) and UV irradiated at 40 J/m^2^ followed by a 2-h recovery, and PLA was performed as described by Makinwa et al. (2020a). Anti-Bid antibody (Mouse monoclonal, Santa Cruz SC-514622) at a dilution of 1:500 mixed with anti-ATR antibody (Rabbit polyclonal, Bethyl Laboratories A300-137A) at a dilution of 1:1,000 was used to determine ATR-Bid interactions. To show ATR–Bid interaction at mitochondria, the cells were probed with a mtHsp70 monoclonal antibody (Mouse monoclonal, Invitrogen MA3-028) at a dilution of 1:50 for at least 1 h at room temperature, washed with PLA wash buffer 2, and incubated with DyLight 488 goat-antimouse IgG secondary antibody (Invitrogen, 35502) at a dilution of 1:400 for 30 min at room temperature. ATR oligomerization among various ATR mutants were monitored by mixing anti-FLAG (Rabbit polyclonal, Gene Tek GTX115043) at a dilution of 1:1,000 with anti-Myc (Mouse monoclonal, Cell Signaling 9B11) at a dilution of 1:4,000. ATR:ATRIP interactions among various ATR mutants were monitored by mixing anti-FLAG (Rabbit polyclonal, Gene Tek GTX115043) at a dilution of 1:1,000 with anti-Myc (mouse monoclonal, Cell Signaling 9B11) at a dilution of 1:4,000. ATRIP dimerization was monitored by mixing anti-HA (Rabbit monoclonal, Invitrogen 2–2.2.14) at a dilution of 1:500 with anti-Myc (Mouse monoclonal, Cell signaling 9B11) at a dilution of 1:4,000. PLA foci were analyzed by one-way ANOVA using GraphPad Prism 5.01 software and are reported as the average ± SD. The paired *t*-test was used to test the significance, and *p* < 0.05 is considered significant.

### *In Vitro* Kinase Activity Assay

The ATR^flox/−^ cells transfected with a plasmid construct of FLAG-ATR (WT), FLAG-ATR (S428A), or FLAG-ATR (P429A) were UV treated as mentioned earlier, and cytoplasmic and nuclear lysates were prepared. Flag-ATR was IPed from cytoplasmic or nuclear extracts. The IP ATR was washed three times with PBS containing 0.05% NP40, followed by a kinase buffer wash [50 mM HEPES (pH 7.5), 150 mM NaCl, 10 mM MgCl2, 10 mM MnCl2, 2 mM DTT, 10% glycerol, 1× protease and phosphatase inhibitors, and 0.5 mM ATP]. The IP ATR was suspended in 20 μL of kinase buffer containing 0.5 mM ATP and 0.5 μg of GST-p53 protein (Boston Biochem, SP-454). The reaction was incubated at 30°C for 30 min and stopped by the addition of SDS-loading buffer. Proteins were separated by 3–8% TA SDS-PAGE and were analyzed by WB using phospho-S15 p53 antibody. IP ATR and the amount of GST-p53 in each sample were confirmed by WB.

### Molecular Modeling

The coordinates of main-chain atoms of the 20 amino acid flanking Ser428-Pro429 bond (10 residues on each side), namely, residues 419 to 438 of ATR, were acquired from the incomplete *trans* conformation ATR structure [PDB code: 5yz0 (Rao et al., 2018)]. Side chain atoms for each amino acid were added by the UCSF Chimera swapaa function (Pettersen et al., 2004). Clashes were examined and removed by experimenting with different rotamers. This generated the initial model of the wild-type ATR peptide in trans conformation. To generate the *cis* conformation, the peptide bond between Ser428-Pro429 was turned approximately 180° and then slightly adjusted to remove local clashes. Further adjustments to the side chains of the peptide were introduced by experimenting with different rotamers of affected residues to remove crowdedness or clashes. This generated the initial model of the wild-type ATR peptide in the *cis* conformation. Single-site mutagenesis of Ser428 to Ala (S428A) and Pro429 to Ala (P429A) in *cis* and *trans* conformation was generated by changing the residue at position 428 and 429 to alanine in the previously mentioned wild-type peptides, respectively. All six peptides were capped by an acetyl group on the N-terminal and by an amide group on the C-terminal to remove the charges and mimic the peptide sitting in the ATR protein.

The N-terminal ATR model that contains residue 2 to 770 was constructed similarly. The coordinates of main-chain atoms of residue 2 to 770 were acquired from *trans* conformation ATR (PDB code: 5yz0). Side-chain atoms for each amino acid were added by the UCSF Chimera swapaa function iteratively by a Python code. Clashes were examined and removed by experimenting with different rotamers. This generated the initial model of the wild-type ATR peptide in *trans* conformation. To generate the *cis* conformation, the peptide bond between Ser428-Pro429 was turned approximately 180° and then slightly adjusted to remove local clashes. In addition, the loops containing residues 351 to 363 and residues 419 to 427 were reconstructed using ModLoop (Fiser et al., 2000) to remove clashes. Both models were capped by an amide group on the C-terminal to remove the effects of the charges, but not capped on the N-terminal to mimic the fragment sitting in the N-terminal of the full-length ATR protein.

### Molecular Dynamics Simulations and Energy Calculation

Hydrogen atoms were added, and FF14SB parameters were assigned to the protein by the tleap module of AMBER for both peptides and N-terminal ATR (Case et al., 2021). Water explicit MD simulations of the six peptides were carried out using the AMBER package. TIP3P explicit water molecules were added around each peptide 18 Å in each direction in a rectangular box, and counter ions were added to neutralize each system. Particle mesh Ewald (PME) was used to calculate the long-range electrostatic interactions, and the nonbonded cutoff was set to 8.0 Å. Each system was equilibrated by a four-step protocol before production MD simulation as previously described (Liu et al., 2011b). Restraint to the protein was removed in the 10 ns equilibrium run. A production trajectory of 400 ns employed the same condition of the final equilibration step, was run for each system. A total of 200 snapshots were collected from the last 200 ns production trajectory for total free energy calculations using the molecular mechanics-generalized Born surface area (MM-GBSA).

Water implicit MD simulations of the two N-terminal ATR were carried out by AMBER package using the Born solvation model for expedited computation. First, the structures were minimized by 500 steps of steepest descent minimization followed by 500 conjugate gradient minimizations with the restraint of 5 kcal/mol/Å^2^ applied to the heavy atoms of the proteins. Salt concentration was set at 0.2 M. The computation of effective Born radii was set at 30 Å. Each system was heated up from 0 to 300 K gradually, with a weak restraint of 2 kcal/mol/Å^2^ on the backbone atoms only. Restraint was then removed in the last 402 ns equilibration run without cutoff for nonbonded interactions. A production trajectory of 20 ns was run for each system using the same condition as the equilibration step. A total of 200 snapshots were collected from the 20 ns production trajectory for total free energy calculations using the molecular mechanics generalized Born surface area (MM-GBSA).

To verify the equilibrium state of each system and perform statistical analysis, total free energies of 200 frames were extracted frame-by-frame using a Python code for all simulations. Two-sided Student’s *t*-test was performed by the R-package 4.1.1 (R Core Team, 2021), and a value of *p* < 0.05 was considered significant.

## Results

### Protein Purification and Chemical Modification of ATR

**FIGURE 1:**
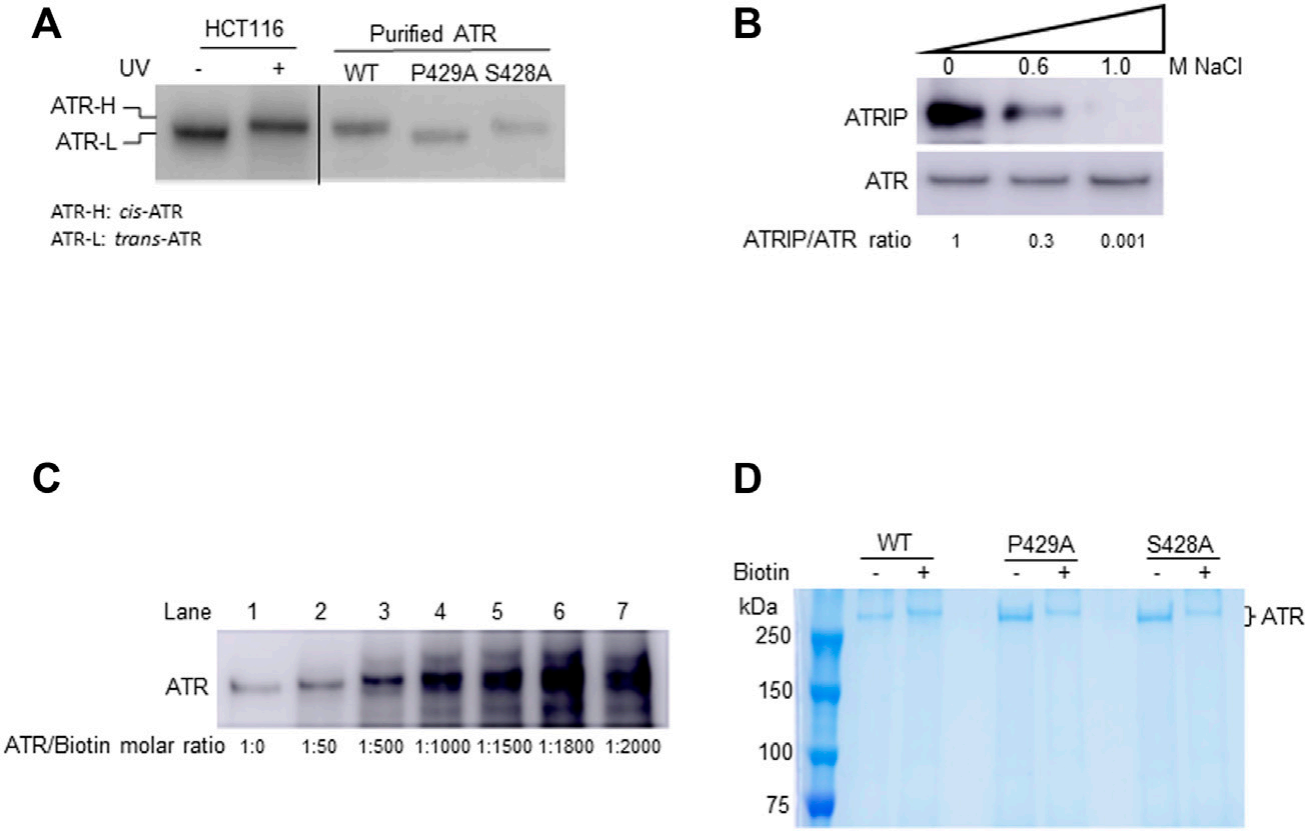
In-solution biotinylation of ATR proteins. **(A)** Recombinant FLAG-ATR WT, P429A, and S428A proteins were purified from HEK293T cells. Purified WT and S428A proteins have the same electrophoretic mobility as endogenous ATR-H purified from the cytoplasmic fraction of UV-treated HCT116 cells, but P429A protein migrates as ATR-L as analyzed by Western blotting in 3–8% gradient SDS-PAGE. **(B)** FLAG-ATR protein was immunoprecipitated and washed with increasing concentrations of NaCl solution to remove bound proteins. ATRIP forms a tight complex with ATR and is efficiently removed by washing with 1M NaCl. The ATRIP/ATR ratios were normalized to the ratio at zero salt concentration. **(C)** Analysis of the mass of ATR-WT after biotinylation using increasing concentrations of sulfo-NHS-LC-biotin on 8% SDS-PAGE. ATR (2 µM) was biotinylated with sulfo-NHS-LC-biotin at different molar ratios and analyzed by Western blotting using ATR antibody. **(D)** 2 µM each of FLAG-ATR WT, P429A, and S428A proteins purified using FLAG beads, run on 8% SDS-PAGE with and without biotin (1,000 molar excess) modification, and visualized by Coomassie staining. Protein bands were excised and digested with trypsin for mass spectrometry analysis.

In unstressed cells, Pin1 isomerizes ATR at p-Ser428-Pro429, converting the *cis*-Ser428-Pro429 amide bond to a *trans*-Ser428-Pro429 bond; this isomerization is dependent on the phosphorylation of Ser428 in the cytoplasm where the *cis-isoform* is the stable form in the absence of Pin1 (Hilton et al., 2015). The former is named *cis*-ATR and the latter, *trans*-ATR. As peptide bonds formed between any pair of the 19 non-proline amino acids adopt the *trans* conformation whereas proline can result in either a *cis*- or *trans*-isomer (Fischer et al., 1984; Fischer and Schmid, 1990; Hinderaker and Raines, 2003). A P429A point mutation was introduced in an ATR construct to ensure a *trans* conformation of the S428-P429A amide bond in ATR protein (ATR-L) (Hilton et al., 2015). In contrast, ATR-S428A protein was predominately *cis*-ATR (ATR-H) in the cytoplasm of cells as the serine to alanine mutation prevents the phosphorylation of ATR-Ser428 necessary for Pin1 isomerization of ATR (Hilton et al., 2015; Makinwa et al., 2020a; Makinwa et al., 2020b). In addition, purified ATR-WT protein from cells was found to be *cis*-ATR after removing Pin1 and all other ATR-interacting proteins through a high salt wash (Figure 1A; Hilton et al., 2015), indicating that wild-type ATR alone is naturally stable in the *cis*-isoform. The same is true for purified ATR-S428A, but purified ATR-P429A is *trans*-ATR (Figure 1A; Hilton et al., 2015).

In this study, recombinant ATR protein containing an N-terminal FLAG tag was overexpressed and purified from HEK293T cells. To obtain relatively pure ATR, FLAG bead-bound FLAG-ATR was washed with a high salt-containing buffer to remove proteins associated with FLAG-ATR including ATRIP 29 Figure 1. Analysis of the samples by Western blotting of a 3–8% gradient SDS gel shows that the purified WT and S428A ATR co-migrated with the ATR-H form, whereas P429A maintains ATR-L (Figure 1A). Table 1 lists different conformational states of ATR observed in cells or after ATR purification from cells (ATRIP or any proteins associated with ATR were removed during ATR purification).

**TABLE 1:** Identified conformational variations of ART WT, S428A, and P429A in cells or after isolation *via* FLAG-tagged immunoaffinity purification and a high salt wash.

	Cytoplasm			Nucleus		
ATR isomer	WT	S428A	P429A	WT	S428A	P429A
In cell	*Cis/trans*	*Cis*	*Trans*	*Trans*	*Trans*	*Trans*
Purified	*Cis*	*Cis*	*Trans*	*Cis*	*Cis*	*Trans*

To determine differences in any surface topology of ATR (WT, P429A, and S428A), purified proteins were chemically modified at their lysine residues using sulfo-NHS-LC-biotin. Sulfo-NHS-LC-biotin derivatives is known to possess an active ester group that reacts specifically with the primary amines of proteins and/or the amino group of lysine residues, thereby forming an amide bond. For each lysine residue, this reaction increases the mass of 339.161 Da. Therefore, we first tested for the molar ratio of sulfo-NHS-LC-biotin to ATR protein. Since ATR has 175 lysine residues, it is expected that under ideal conditions all these residues can be biotinylated. This, however, assumes that all lysine residues are accessible to the biotin moiety, which is unlikely considering the tertiary structure of a protein. Nevertheless, if all the 175 lysine residues are biotinylated, the calculated mass shift of the protein would be 59.35 kDa. As shown in Figure 1C, an increase in the concentration of the biotinylating reagent lead to an increase in the mass of the protein as demonstrated by the band shifts in SDS-PAGE (Figure 1C, lanes 1–4) up to a maximum of 1000-fold molar excess of biotin (Figure 1C, lane 4). Further increase in concentration to 1500-fold molar excess does not result in any significant increase in the mass of the protein (Figure 1C, lane 5). No further increase in mass also indicates that there is a stable tertiary structure of ATR under the experimental modification conditions. Therefore, all three forms of affinity-purified FLAG-ATR proteins were modified using a 1000-fold molar excess of sulfo-NHS-LC-biotin and re-purified on SDS-PAGE (Figure 1D) before tryptic digestion and MALDI-TOF analysis.

### Surface Topology Analysis of Wild-Type, P429A (*trans*), and S428A (*cis*) ATR With the Biotinyl-Lysine Method and Mass Spectrometry

**FIGURE 2:**
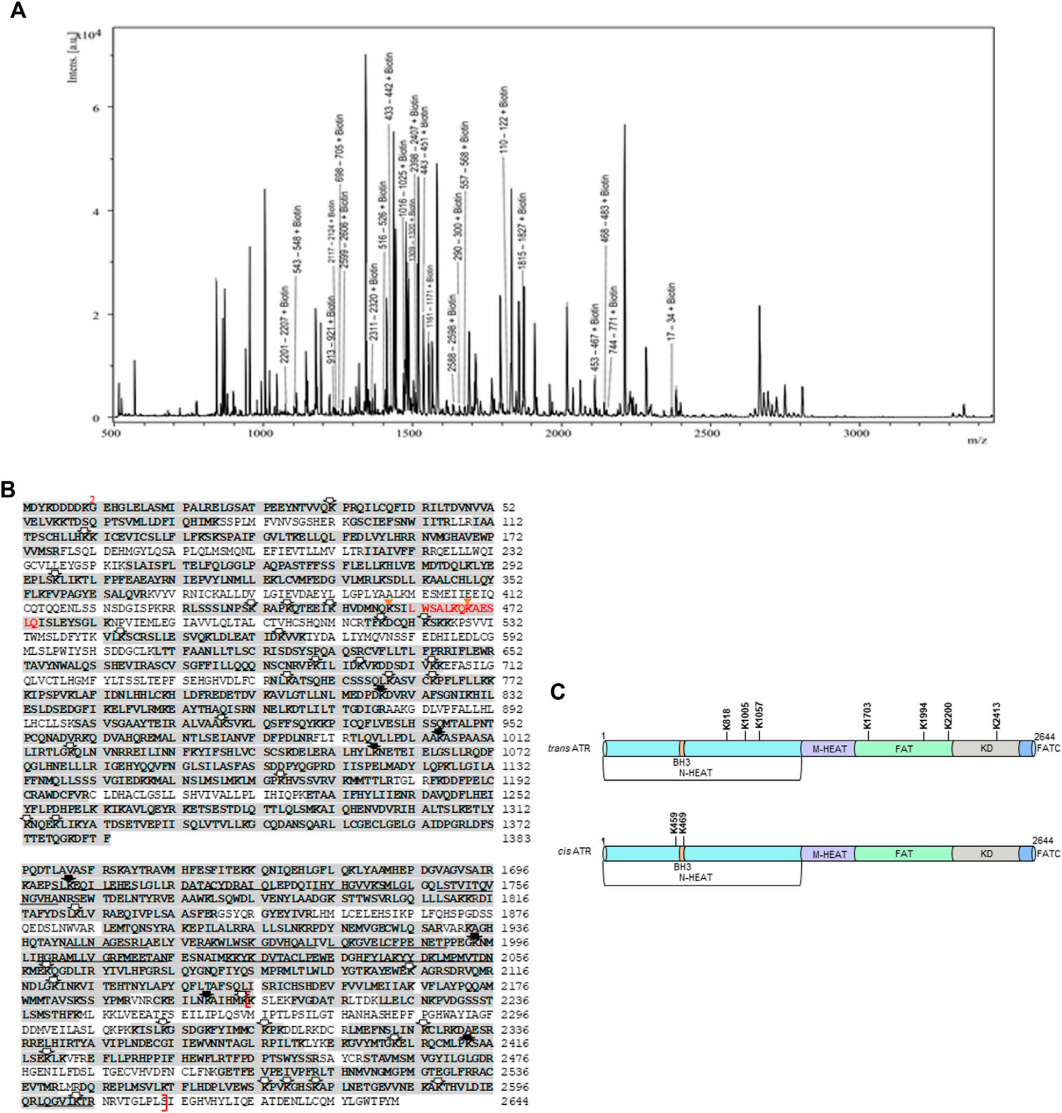
MALDI-TOF and nano LC-MS/MS analysis of biotin-modified *cis*- and *trans*-ATR. **(A)** Typical MALDI-TOF mass spectrum of peptide fragments resulting from trypsin digestion of biotin-modified *cis-*ATR. **(B)** Sequence of N-HEAT, FAT, KD, and FATC domains of FLAG-tagged ATR protein. The initial methionine is part of the FLAG tag, and the first amino acid corresponding to ATR is marked as 2. Amino acid sequences corresponding to tryptic peptide fragments detected by MALDI-TOF and nano LC-MS/MS are indicated by the shading. The lysine residues modified by NHS-biotin treatment for all *trans*- and *cis*- *and WT* ATR are indicated by open arrows, whereas unique biotin-modified lysine residues were observed for *trans*-ATR (solid arrow) and *cis*- and WT ATR (gold triangle). The BH3 domain (aa461-474) is designated by red color, and the kinase domain (aa2206-2615) is enclosed in red brackets. **(C)** Summary of unique and differential biotin-modified lysine residues between *trans*- and *cis*-ATR identified by MALDI-TOF and nano LC-MS/MS in the context of the ATR structure. N-HEAT, N-terminal HEAT repeats; BH3, BH3-like domain; M-HEAT, middle HEAT repeats; FAT, FRAP, ATM, TRRAP domain; KD, kinase domain; FATC, FAT C-terminal domain.

Chemical modification coupled with mass spectrometry has been used to probe the surface topology of proteins (Bennett et al., 2000), including proteins interacting with ATR (Shell et al., 2009). To identify biotin-modified lysines, tryptic peptides were subjected to matrix-assisted laser desorption/ionization-time-of-flight mass spectrometry (MALDI-TOF). MALDI-TOF, without chromatographic separation of the peptides, a series of peaks with a mass-to-charge ratio (m∕z) corresponding to the tryptic peptides unfolds in a single-mass spectrum. Because the sample is known in this case, it is possible to identify the ATR peptide corresponding to each peak by calculating all possible peptide masses for tryptic digestion of ATR, including biotin modification of lysines. We determine a probability-based score, called the Ascore, which measures the probability of correct biotin-modified site localization based on the presence and intensity of site-determining ions in MS/MS spectra (Supplementary Table S1). A total of 185, 195, and 191 tryptic peptides with 41, 46, and 41 biotinylated lysines were identified, respectively, for WT, P429A, and S428A ATR proteins by peptide mass matching after MASCOT search. Figure 2A depicts a representative mass spectrum for tryptic fragments of the biotin-modified WT ATR protein. Monoisotopic resolution of the peaks allowed us to rightfully identify the tryptic fragments of ATR. Figure 2B illustrates the peptide fragments (bold sequences) and biotinylated lysines identified by MALDI-TOF as well as high-resolution nano LC-MS-MS analysis for ATR (WT, P429A, and S428A). We have identified seven unique biotin-modified lysine residues at K818, K1005, K1057, K1703, K1994, K2200, and K2413 for ATR P429A (ATR-L) whereas two unique biotin-modified lysines at K459 and K469 were observed for WT and S428A (ATR-H) Figure 2C.

### Identification of Conformational Change of the tBid-Binding Domain of *cis/trans*-ATR Isoform

**FIGURE 3:**
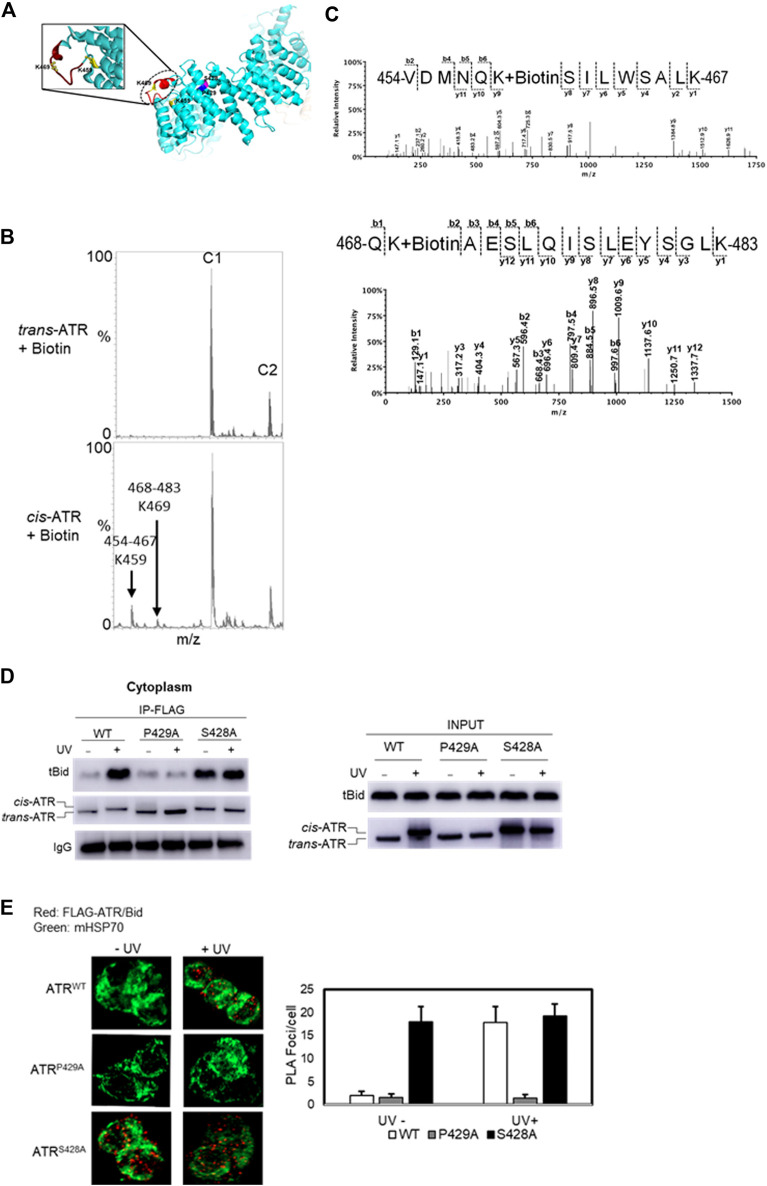
Effect of *cis/trans* conformation of ATR on ATR–tBid interaction. **(A)** In ribbon diagram of ATR (PDB code 5YZ0, Rao et al., 2018), the positions of S428A and P429A mutant sites on the ATR (chain colored with cyan) structure are colored with magenta and blue, respectively. tBid interacting with the BH3 domain of ATR is shown by red color marked with a dotted circle. Biotin-modified K459 and K469 amino acids of *cis*-ATR are displayed by ball and stick. **(B)** Representative segment of MALDI-TOF data showing that K459 and K469 were readily modified with sulfo-NHS-LC-biotin in cis-ATR but were significantly protected from the modification in trans-ATR. Unmodified peaks C1 and C2 serve as controls. **(C)** Mass spectrum of identified biotin-modified Lys-459 amino acid of peptide 468QKAESLQISLEYSGLK483 and K469 amino acid of peptide 454VDMNQKSILWSALK467 of *cis-*ATR. A 147.1 Da mass increase of precursor ion and 339.1 Da increase in mass between b1, b2 and y2, y3 ions is observed for K459 and K469, respectively. **(D)** tBid interacts with the *cis* form of ATR (ATR-H). ATR^flox/-^ cells were transfected with a plasmid construct of FLAG-ATR (WT), FLAG-ATR (S428A), or FLAG-ATR (P429A). *In vitro* pull-down of purified tBid protein was carried out with FLAG bead-bound ATR proteins isolated from the cytoplasmic fractions of UV- or mock-treated ATR^flox/-^ cells. **(E)** Duolink PLA demonstrates that *cis*-ATR interacts with proapoptotic protein Bid. Focus stacking reveals that the UV-induced ATR–Bid interaction predominately occurs outside of the nucleus. Foci formation per cells was calculated considering an average of 50 cells, and each experiment was performed in triplicate. The bar graph represents a statistical analysis of the PLA images.

ATR can form *cis or trans* isomers in the cytoplasm depending on Pin1, which can isomerize cis-ATR to trans-ATR. The cis-ATR, likely containing an exposed BH3 domain versus the unexposed BH3 domain in trans-ATR, is antiapoptotic at mitochondria by binding to tBid, thereby forbidding the activation of pro-apoptotic Bax. We reported previously that the BH3-like domain (aa462–474, Figure 3A) of ATR was required for the ATR–tBid interaction (Hilton et al., 2015). Our data showed that the P429A mutant predominantly forms the trans (ATR-L) isoform, whereas FLAG affinity-purified WT and S428A ATR mutant isoforms migrate as the cis (ATR-H) isoform (Figure 1A). Interestingly, mass footprinting with biotin modification revealed that WT and S428A mutant (cis-ATR) showed a conformational change over the BH3 domain where K459 and K469 were accessible to biotin modification (Figures 3B,C), while the P429A mutant (trans-ATR) had no such modifications. To confirm the mass spectra data, we performed a pull-down assay by incubating purified tBid protein (R & D Systems) with FLAG-immobilized ATR from cytoplasmic fractions of UV+/- treated ATRflox/− cells transfected with a plasmid construct of FLAG-ATR (WT), FLAG-ATR (S428A), or FLAG-ATR (P429A) (Figure 3D). The data indicate that UV irradiation led to the cis-ATR formation in the cytoplasm of cells transfected with ATR-WT construct, while ATR-P429A remains as trans-ATR and ATR-S428A as cis-ATR regardless of UV, as previously reported (Hilton et al., 2015). Furthermore, Figure 3D shows a very dramatic increase of tBid protein pull-down by cis-ATR rather than by trans-ATR. We also observed that in the cells the cis-ATR (ATR-S428A)–tBid interaction occurred at mitochondria regardless of UV (Figure 3E), while as a control, the cis-conformation of ATR-WT induced by UV led to a dramatic increase of cis-ATR–tBid interaction (Figure 3E). In contrast, trans-ATR (ATR-P429A) could not interact with tBid. These results show that in the presence or absence of UV irradiation tBid is associated with cis-ATR but not trans-ATR.

### Effect of *cis/trans* Conformational Change on ATR Dimerization

ATR dimerizes through three symmetrically arranged intermolecular contacts between the C-terminal ATR–ATR interface and also through the N-terminal ATR–ATRIP interface and the coil–coil domain of ATRIP–ATRIP dimerization ATRIP (dimer–dimer) (Rao et al., 2018). The C-terminal regions involved in ATR dimer formation are highly conserved, supporting their significance for ATR function (Rao et al., 2018). We have investigated the dimerization domain of *cis/trans* ATR through mass footprinting. Peptides that are involved in ATR dimerization were identified and are listed in Table 2.

**TABLE 2:** Identified peptides involve in ATR dimerization for ATR WT and mutant P429A and S428A proteins.

*Trans*-ATR	*Cis*-ATR
1696 KAEPSLKEQILEHESLGLLR 1717	1696 KAEPSLKEQILEHESLGLLR 1717
1716 DATACYDR 1725	1716 DATACYDR 1725
1724 AIQLEPDQIIHYHGVVK 1742	1724 AIQLEPDQIIHYHGVVK 1742
1741 SMLGLGQLSTVITQVNGVHANR 1764	1741 SMLGLGQLSTVITQVNGVHANR 1764
1933 AGHHQTAYNALLNAGESR 1952	1933 AGHHQTAYNALLNAGESR 1952
1958 AKWLWSK 1967	1958 AKWLWSK 1967
1966 GDVHQALIVLQK 1979	1966 GDVHQALIVLQK 1979
1978 GVELCFPENETPPEGkNMLIHGR 2002	1978 GVELCFPENETPPEGK 1995, 1994 NMLIHGR 2002
2001 AMLLVGR 2009	2001 AMLLVGR 2009
2008 FMEETANFESNAIMK 2024	2008 FMEETANFESNAIMK 2024
2023 YKDVTACLPEWEDGHFYLAK 2044	2023 YKDVTACLPEWEDGHFYLAK 2044
2043 YYDKLMPMVTDNK 2057	2043 YYDKLMPMVTDNK 2057
2598 LQGVIkTR 2607	2598 LQGVIkTR 2607

*k-biotin-modified lysine.

Mass footprinting data can identify nearly all peptides involved in ATR dimerization for ATR (WT), P429A, and S428A mutant proteins. By use of mass footprinting, we have identified 14 peptides (all unmodified except two) at the dimeric interface for ATR P428A mutant, whereas WT and S428A mutant dimeric interface consists of 15 unmodified peptides (all unmodified except one).

**FIGURE 4:**
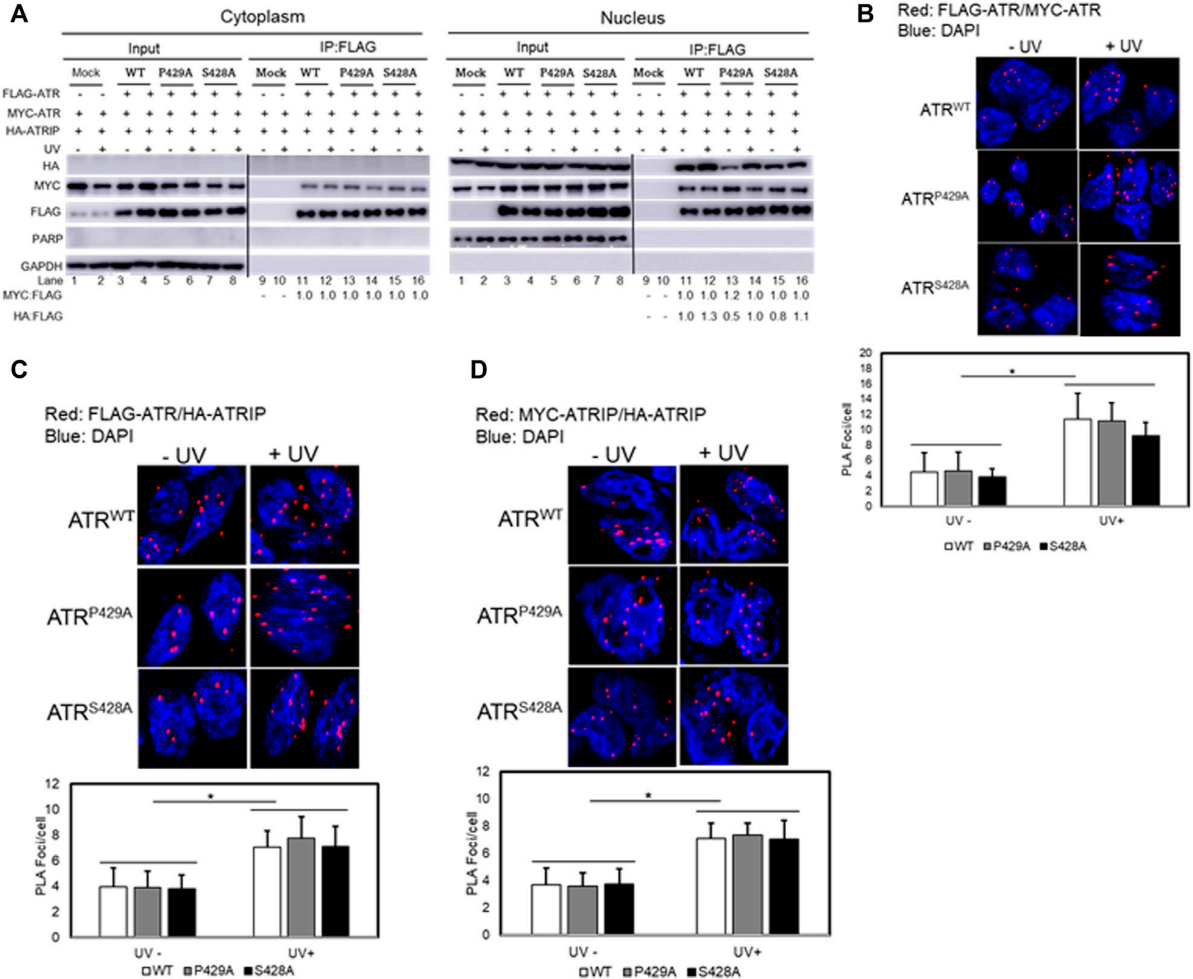
Effect of *cis/trans* isomerization on ATR oligomerization and ATR–ATRIP complex formation. **(A)** Oligomerization of *trans-* and *cis-*ATR was analyzed by co-expressing vector constructs encoding FLAG-ATR, Myc-ATR, and HA-ATRIP transfected into the ATR^flox/−^ cells followed by UV+/− treatments. ATR oligomerization was assessed by co-immunoprecipitation after cellular fractionation. Interestingly, no ATRIP occurred in the cytoplasm as compared to ATRIP in the nucleus. The Myc/FLAG ratios were normalized to the ratio at lane 11 (cytoplasm fraction) and HA/FLAG at lane 11 (Nuclear fraction). **(B)** PLA revealing a direct interaction between FLAG-ATR and Myc-ATR in ATR^flox/−^ cells co-transfected with FLAG-ATR and Myc-ATR plasmids. The interaction was enhanced after UV irradiation at 40 J/m^2^. A DAPI-staining overlay shows the location of the nuclei. **(C)** ATR/ATRIP complex formation was monitored using PLA by co-transfecting FLAG-ATR WT, P429A, or S428A mutant with Myc-ATRIP in ATR^flox/-^ cells. **(D)** ATRIP oligomerization in the presence of FLAG-ATR WT, P429A, or S428A mutant proteins. ATR^flox/-^ cells were co-transfected with Myc- and HA-ATRIP plus FLAG-ATR WT, P429A, or S428A constructs followed by UV irradiation. Oligomerization of Myc- and HA-ATRIP was monitored by PLA. Foci formation per cells was calculated considering an average of 50 cells, and each experiment was performed in triplicate. The bar graphs represent the statistical analysis of the PLA images. * stands for the *p*-value < 0.01.

We next examined whether *cis* and *trans* isoforms of ATR were oligomers in cells. ATR oligomerization was analyzed by co-expressing FLAG- and Myc-tagged ATR proteins in UV+/− treated ATR^flox/−^ cells co-transfected with plasmid constructs of FLAG-ATR (WT), FLAG-ATR (S428A), or FLAG-ATR (P429A) and Myc-ATR (WT), Myc-ATR (S428A), or Myc-ATR (P429A), followed by assessing ATR oligomerization by co-IP after fractionation. Immunoprecipitation of FLAG-ATR using FLAG antibodies (Figure 4A) demonstrates that ATR does form oligomeric complexes in cells. FLAG antibodies did not immunoprecipitate any Myc-ATR when FLAG-ATR was not expressed (Figure 4A cytoplasmic fraction Lane 9 and 10 and nuclear fraction Lane 9 and 10). A significant reduction in the amount of ATR-P429A-IPed HA-ATRIP in the absence of UV is likely due to the experimental variation in efficiency of HA-ATRIP construct transfection into cells. Oligomerization of *cis/trans* isoforms of ATR was further confirmed by the proximity ligation assay method by co-expressing FLAG- and Myc-tagged ATR in UV+/− treated ATR^flox/−^ cells (Figure 4B).

### Effect of *cis/trans* Conformational Change on ATR–ATRIP Association

The conformational flexibility of ATR allows ATRIP to properly lock up the N-termini of the two ATR monomers *via* the C-termini of two ATRIP monomers toward favorable ATR–ATRIP complex formation and functional diversity (Cortez et al., 2001; Ball et al., 2005; Falck et al., 2005). To understand the effect of *cis/trans* isomerization on ATR–ATRIP association, UV+/− treated ATR^flox/−^ cells were transfected with vectors encoding FLAG-ATR, Myc-ATR, and HA-ATRIP. ATR–ATRIP association was determined by FLAG-immunoprecipitation after cellular fractionation. As shown in Figure 4A, nuclear ATR formed a complex with ATRIP and UV irradiation enhanced the complex formation. Interestingly, little or no ATRIP was found in the cytoplasm as compared to ATRIP in the nucleus (Figure 4A). Thus, there was little or no cytoplasmic ATR–ATRIP complex formation. The nuclear ATR–ATRIP association for *cis/trans* ATR isoforms was confirmed further by the proximity ligation assay (Figure 4C).

The ATRIP–ATRIP association was assessed using the proximity ligation assay under the influence of ATR *cis/trans* isoforms. UV+/− treated ATR^flox/−^ cells were co-transfected with FLAG-ATR, Myc-ATRIP, and HA-ATRIP. ATRIP oligomerization under the influence of ATR (WT, P429A, and S428A) was monitored by fluorescence microscopy (Figure 4D). Data suggest that ATRIP–ATRIP foci formation increased similarly by UV treatment for all ATR isoforms, which signifies the formation of stable ATR–ATRIP complexes.

### Conformational Changes Around the Kinase Domain Among ATR Mutants

Recently reported cryo-electron microscopy structural data of the human ATR–ATRIP complex reveals key components of the kinase domain related to ATR function (Figure 3A) (Rao et al., 2018). The PIKK regulatory domain (PRD, residues 2,483–2,597) of ATR and ATRIP (C-terminal coiled-coil domain) are both crucial for TopBP1-mediated activation. Mutation K2589E does not impair basal kinase activity of ATR but largely diminish its activation by TopBP1 (Mordes et al., 2008). Previous studies also showed that ATR undergoes autophosphorylation on residue T1989, which recruits TopBP1 for the activation of ATR (Liu et al., 2011a; Nam et al., 2011). Structural analysis reveals that residue T1989 is located on the surface of the FAT domain and is unlikely to gain access to any of the two catalytic pockets within the same ATR–ATRIP complex, suggesting *trans*-mode autophosphorylation. Since residues T1989 and K2589 both contribute to TopBP1-mediated activation of ATR, it is comprehensible that TopBP1 binds to ATR along with the dimer interface. Residue K2589 stabilizes the substrate entry groove of the kinase domain followed by a loop involved in intermolecular contacts and plays a role in the regulation of substrate entry. In sum, TopBP1 might stimulate ATR–ATRIP kinase activity by facilitating substrate access to the catalytic cavity or inducing conformational changes of the kinase domain to favor the catalytic reaction.

**FIGURE 5:**
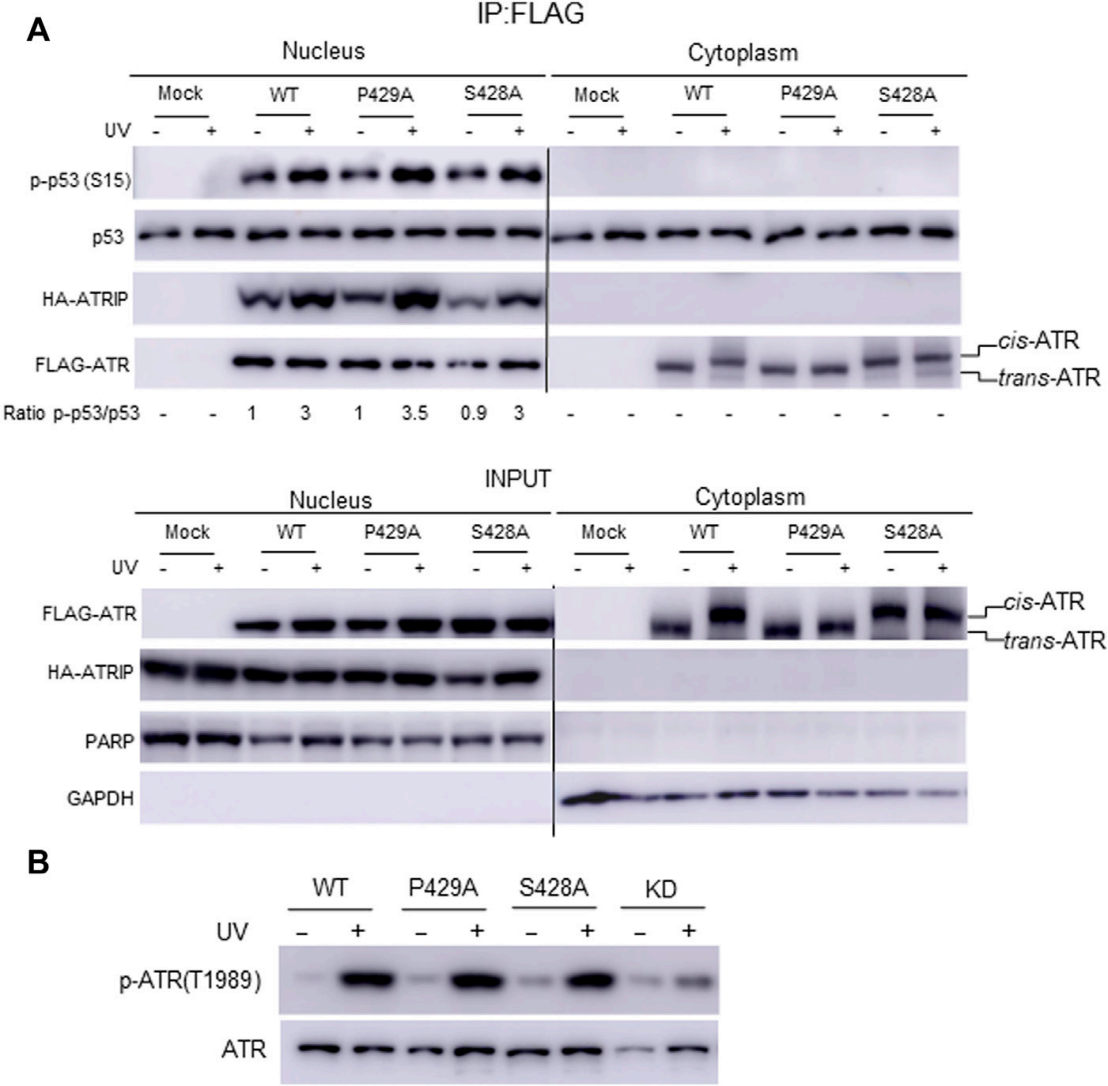
Effect of *cis/trans* conformation of ATR on its kinase activity. **(A)** *In vitro* phosphorylation of purified GST-p53 was carried out to assess the checkpoint kinase activity of IPed ATR from the cytoplasmic and nuclear fractions of ATR^flox/−^ cells expressing co-transfected FLAG-ATR WT, mutants P429A, or S428A plus Myc-ATRIP constructs. GST-p53 phosphorylation was monitored by Western blot using phospho-S15 p53 antibody. **(B)** Phosphorylation of ATR on T1989 was monitored to confirm the checkpoint kinase activity of ATR WT, S428A, and P429A mutant proteins in ATR^flox/−^ cells after transfection with the respective plasmid construct. The nuclear fraction was collected from UV+/− irradiated cells after 2 h of recovery.

Our mass footprinting data uncover kinase domain assembly between ATR WT as well as the S428A and P429A mutants. All these proteins show the same peptide fragmentation pattern along the catalytic loop and the activation loop of ATR (Rao et al., 2018). Residue K2859, which is important to maintain conformation at the substrate entry groove, was found to be biotin-modified for ATR WT as well as the S428A and P429A mutants. Since nuclear ATR plays an important role in DNA damage-induced checkpoint activity in association with ATRIP, we examined the checkpoint kinase activity of cytoplasmic ATR. A previous report provides evidence that phosphorylation at Ser15 is a critical event in the upregulation and functional activation of p53 during cellular stress and ATR regulates phosphorylation of Ser15 in DNA-damaged cells (Tibbetts et al., 1999). To test the phosphorylation status of p53(Ser15), ATR WT, S428A, and P429A mutant plasmid constructs were transfected into ATR^flox/−^ cells. FLAG-ATR was IPed from cytoplasmic and nuclear fractions of UV+/− irradiated cells after 2-h of recovery and subjected to an *in vitro* kinase assay for phosphorylation of p53(Ser15) by ATR. All three proteins from the nucleus show the equivalent kinase activity toward p53 (Figure 5A), consistent with nuclear ATR, either WT, S428A, or P429A being *trans*-ATR (Hilton et al., 2015; Makinwa et al., 2020a) Table 1. In contrast, upon UV irradiation p53 was not phosphorylated by cytoplasmic ATR, regardless of *cis* or *trans* conformation, due to the lack of ATRIP (Figure 5A).

Interestingly, a peptide fragmentation pattern difference is observed between the ATR-P429A mutant with ATR-WT and ATR-S428A mutant. The trypsin digestion-protected peptide ^1978^(K)GVELCFPENETPPEGK(Biotin)NMLIHGR(A)^2002^ with a biotin-modified K1994 is observed in ATR-P429A (Table 2). This K1994 is in very close proximity to T1989, which is at the ATR auto-activation site. In contrast, K1994 is not biotin modified in either WT or S428A ATR, resulting in two tryptic peptides ^1978^(K)GVELcFPENETPPEGK(N)^1995^ and ^1994^(K)NMLIHGR(A)^2002^. This implies that there could be a conformational alteration between *cis*-ATR (WT or S428A) and *trans*-ATR (P429A), which might affect the kinase activity of ATR. However, this effect may occur only in the cytoplasm as in the nucleus when both ATR WT and S428A are *trans*-ATR and should have the same conformation as ATR P429A. Indeed, the results in Figure 5 indicate that the three ATR proteins, WT, S428A, and P429A, have the equivalent kinase activity in the nucleus.

Previous studies showed that ATR is transformed into a phosphorylated state after DNA damage and that a single autophosphorylation event at Thr1989 is crucial for ATR activation. Phosphorylation of Thr1989 relies on RPA, ATRIP, and ATR kinase activity. To test the autophosphorylation status at Thr1989, ATR WT, S428A, and P429A mutant plasmid constructs were transfected in ATR^flox/−^ cells, and the nuclear fraction was collected from UV+/− irradiated cells after 2 h of recovery. Autophosphorylation at Thr1989 was monitored using an anti-Thr1989 phosphor ATR antibody. ATR KD version was also included as a negative control. The WB shows that there is no significant difference in autophosphorylation at Thr1989 for nuclear ATR WT and S428A mutant compared with the P429A mutant (Figure 5B). This is consistent with the fact that all three ATRs are always in the *trans* conformation in the nucleus. Recruitment of ATR–ATRIP to RPA-ssDNA leads to the congregation of ATR–ATRIP complexes and promotes Thr1989 phosphorylation in trans-ATR 24.

### Structural Characterization by Molecular Modeling and Computational Biology

To understand the proline isomerization of ATR at Ser428-Pro429 from the standpoint of free energies, we conducted molecular modeling and molecular dynamics (MD) simulations using the AMBER package. Due to the huge size of ATR and the incompleteness of the experimentally determined ATR structure (Rao et al., 2018), we derived 20-aa peptide with S428-P429 flanked by 9-aa on each side (NLSSNSDGISPKRRRLSSSL) in both *cis* and *trans* conformations, performed water explicit MD simulation, and calculated the total free energies for both conformations using the MM-GBSA approach. Because small peptides do not fold like protein and form rigid structures, root mean square deviation (RMSD) of the main chain atoms are greater than 3.5 Å during the simulations. To verify each system has reached its equilibrium status, we developed a Python code and extracted total free energies frame-by-frame from the MM-GBSA calculation. We then plotted and examined the data. The energies are steady and flat without further declining (Supplementary Figure S1A), suggesting each system is well-equilibrated. As shown in Table 3, the *cis* conformation of wild-type ATR is energetically more favorable than *trans* conformation with a total free energy of −759 kcal/mol vs. −756 kcal/mol. On the contrary, in the case of P429A mutant, the *cis* conformation is less stable than *trans* with a total free energy of −759 kcal/mol compared with −776 kcal/mol. This is consistent with previous findings that peptide bonds of all other 19 amino acids not involving proline favor the *trans* conformation (Zimmerman and Scheraga, 1976). However, when S428 is substituted by alanine, this trend reversed with *cis* conformation is more favorable than that with *trans* (total free energy −741 vs. −738 kcal/mol). Moreover, the aforementioned pattern of total free energies of the six types of peptides reoccurred in our duplicated runs (Supplementary Table S2). Therefore, the computational results fully agree with the experimental observations that *cis* conformation dominates wild-type and the S428A mutant, while *trans* conformation is preferred in the P429A mutant.

**TABLE 3:** Calculated total free energies and components of wild-type and mutant ATR peptides (WT: NLSSNSDGIS428P429KRRRLSSSL; S428A: NLSSNSDGIA428P429KRRRLSSSL; and P429A: NLSSNSDGIS428A429KRRRLSSSL).* All values are reported in kcal/mol. *p*-values are from two-sided Student’s t-test.

Energy terms*	*Cis*-WT		*Trans-*WT		*Cis*-P429A		*Trans-*P429A-		*Cis*-S228A		*Trans-*S428A	
	Mean	S.D.	Mean	S.D.	Mean	S.D.	Mean	S.D.	Mean	S.D.	Mean	S.D.
Bond	64.39	6.94	62.77	6.61	61.76	6.44	60.92	6.72	62.73	6.98	63.02	6.70
Angle	165.05	9.79	158.48	9.46	153.17	10.09	149.53	9.70	158.96	10.55	157.95	10.28
Dihed	256.48	6.35	256.78	7.80	251.04	7.29	252.43	6.90	255.35	7.47	255.74	7.51
VDW	−95.65	7.62	−95.00	8.70	−84.75	10.95	−95.02	7.49	−91.47	9.09	−90.10	9.62
ELE	−943.35	32.35	−85.37	45.31	−875.37	50.25	−902.93	41.59	−911.64	48.52	−859.37	40.87
1–4 VDW3	65.9	3.85	66.04	3.73	64.93	3.51	66.04	3.72	66.47	3.92	65.92	3.58
1–4 EEL	141.06	14.73	144.19	22.51	151.46	21.00	159.78	22.39	151.92	21.82	154.95	20.92
G-Gas	−346.09	33.38	−292.12	39.03	−277.76	47.53	−309.25	35.29	−307.68	43.42	−251.90	40.19
G-Solv	−413.38	25.45	−464.22	32.32	−481.49	40.36	−466.79	27.20	−433.77	33.69	−486.40	33.64
Total	−759.47	15.53	−756.34	15.12	−759.26	15.85	−776.04	15.63	−741.45	15.85	−738.29	14.99
*p*-value	0.042				<2.2e-16				0.042			

* All values are reported in kcal/mol. *p*-values are from two-sided Student’s t-test.

To understand if *cis*-ATR is energetically more favorable than *trans*-ATR beyond the local peptide level, we built the *cis* and *trans* models comprising residues 2–700 and performed water implicit MD simulation. Both *cis*- and *trans*-ATR are composed of helices and loops that are loosely packed, and both structures are dynamically fluctuating tremendously as indicated by the main chain RMSD value. To ensure each system has reached its equilibrium status, we extracted the total free energies frame-by-frame from the MM-GBSA calculation and examined them to be flat and steady without further declining (Supplementary Figure S1B), suggesting that the system is well-equilibrated. As shown in Table 4, the *cis* conformation of wild-type ATR is energetically more favorable with a total free energy of −14,666 kcal/mol vs. −14,531 kcal/mol in *trans* conformation. This is also true in duplicated runs in which *cis*-ATR has a total free energy of −14,747 kcal/mol compared with −14,509 kcal/mol of that of *trans*-ATR.

**TABLE 4:** Calculated total free energies and components of N-ATR (residue 2–770) in *cis* and *trans* conformation.

Energy terms*	Initial runs				Duplicated runs			
	*Cis*-AA770		*Trans*-AA770		*Cis*-AA770		*Trans*-AA770	
	Mean	S.D.	Mean	S.D.	Mean	S.D.	Mean	S.D.
Bond	2,481.41	42.17	2,487.54	45.03	2,482.17	40.33	2,487.15	41.84
Angle	6,581.86	63.94	6,570.12	70.70	6,575.29	61.87	6,558.83	63.29
Dihed	9,774.51	39.64	9,766.92	38.92	9,803.42	40.60	9,797.98	42.37
VDW	−5,650.77	54.81	−5,358.51	52.50	−5,753.14	61.85	−5,387.97	50.02
ELE	−53,120.93	236.72	−52,319.35	209.00	−53475.41	213.20	−52,892.09	257.08
1–4 VDW	2,810.94	23.54	2,822.22	22.24	2,828.29	22.55	2,816.45	22.17
1–4 EEl	33,121.40	81.34	33,206.63	93.87	33,138.63	77.70	33,295.76	81.14
G-Gas	−4,001.89	234.72	−2,854.43	207.83	−4,410.75	219.21	−3,306.88	242.81
G-Slov	−10,663.86	192.67	−11,676.44	164.42	−10,336.15	183.02	−11,202.01	207.52
Total	−14,665.75	86.90	−14,530.87	91.30	−14,746.90	92.74	−14,508.89	85.09
*p*-value	<2.2e-16				<2.2e-16			

## Discussion

DNA damage checkpoints and apoptosis are two dominant pathways of the DNA damage response (DDR). Moderate DNA damage activates checkpoints, leading to cell cycle arrest and DNA repair. Checkpoint activation appears to be in sync with the suppression of apoptosis, as eventually checkpoints will subside, and normal cell cycling will resume after completion of DNA repair. To this end, a balance between the levels of ATR-H (*cis*-ATR), an antiapoptotic protein, and ATR-L (*trans*-ATR), a DNA damage checkpoint kinase, is regulated in the cytoplasm. Pin1 is central to the regulation of this balance since it catalyzes the phosphorylation-dependent isomerization that converts *cis*-ATR to *trans*-ATR in the cytoplasm (Hilton et al., 2015; Makinwa et al., 2020a; Makinwa et al., 2020b). The proper balance ensures a functional and effective DNA damage response as the protection of cells from apoptosis is essential for the activities of cell cycle checkpoint arrest and DNA repair. The presented data demonstrate a structure–function mechanism by which the two pathways may work in a coordinated manner in DDR.

**FIGURE 6:**
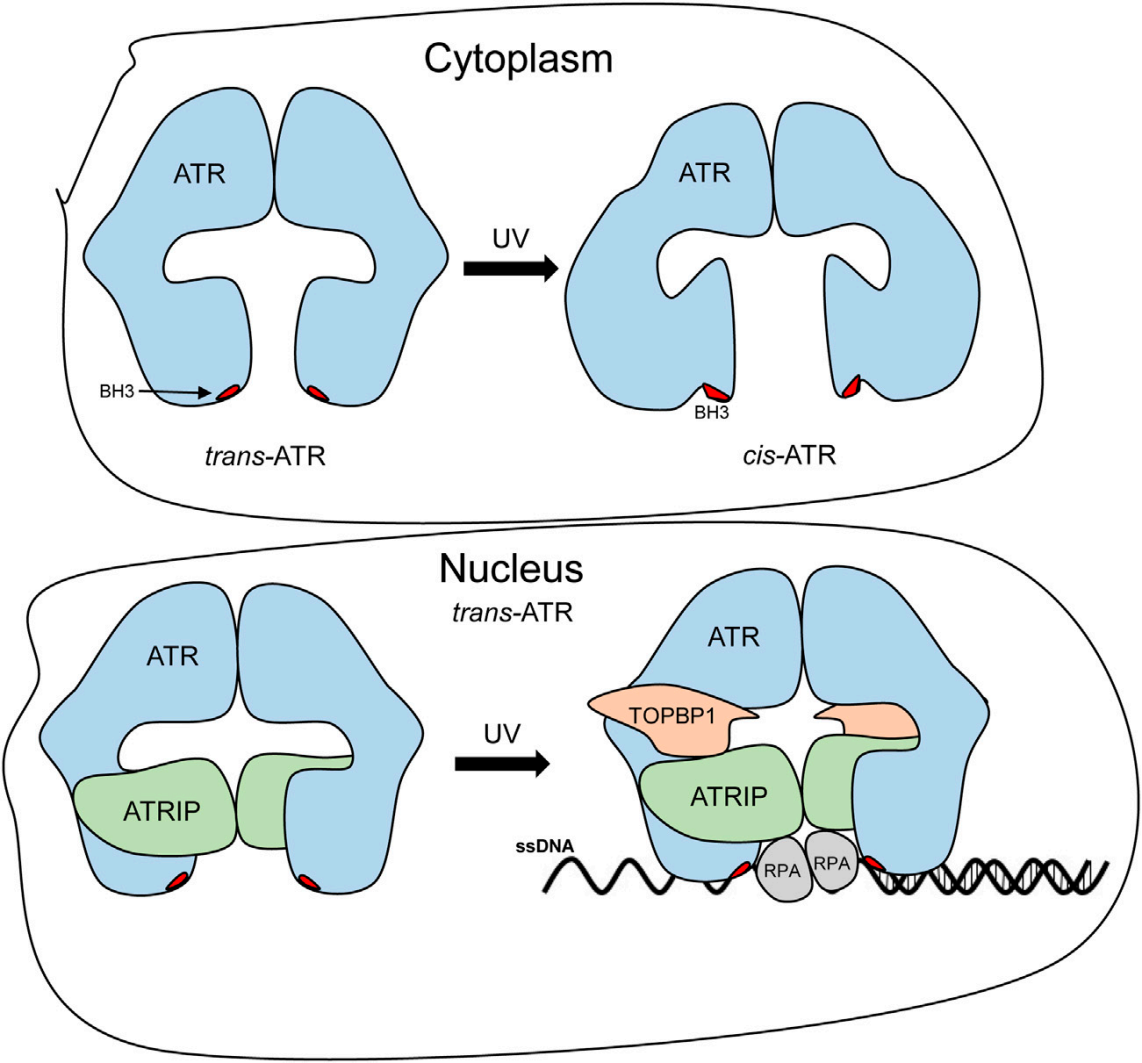
Proposed prolyl isomerization-mediated conformational changes and subcellular compartment-specific functions of ATR. Mass footprinting revealed changes in the surface topology of ATR in the cytoplasm and the nucleus. After UV irradiation, cytoplasmic ATR undergoes multiple conformational changes that open up the BH3 domain and allow tBid–ATR interaction, whereas, in the nucleus, UV irradiation enhances the ATR–ATRIP association, which may help retain the *trans-*ATR conformation and allow the ATR–ATRIP complex to interact with RPA-bound ss-DNA and the subsequent binding of other ATR activators (like TOPBP1), further enhancing ATR kinase activity.

In this study, we demonstrated and confirmed that *cis*-ATR and *trans*-ATR are structurally different, and ATR (purified WT) is naturally stable in *cis* conformation. This is confirmed by our molecular modeling of the 20-aa peptide of ATR with Ser428-Pro429 in the middle of the sequence, and the N-terminal fragment consists of residues 2 to 770, revealing that *cis* conformation is significantly more energetically favorable than *trans* conformation in this wild-type ATR (Table 3, 4). As summarized in Figure 6, the UV-induced *trans*-to-*cis* isomeric conversion in the cytoplasm results in a conformational change in the N-terminal and middle regions of ATR, likely exposing the BH3-like domain. Exposure of the BH3-like domain confers ATR a mitochondria-specific antiapoptotic function. Interestingly, cytoATR shows little checkpoint kinase activity, most likely due to the absence of ATRIP in the cytoplasm (Figure 5A). In contrast, ATR in the nucleus is known to complex with ATRIP, which renders checkpoint activity. Nuclear ATR remains predominately in the *trans*-ATR form (Figure 5A). Knockdown ATRIP even does not change *trans*-ATR conformation in the nucleus (Hilton et al., 2015), implying complex formation with other ATR-interacting proteins and/or chromatins might energetically favor *trans*-ATR in the nucleus to prevent ATR from forming *cis*-ATR. Other speculative possibilities also might play a role in *cis*-to-*trans* conversion in the nucleus such as nuclear transporter proteins. These transporter proteins recognize nuclear import signal (NLS) sequences and can interact with nucleoporins to help NLS-containing proteins reach the nucleus through nuclear pore complexes (NPCs). Nuclear transporter proteins recognize sequences starting with a proline (P) and are followed by an amino acid sequence containing three to five basic residues (Lu et al., 2021). The ATR-predicted nuclear transport signal resides within the isomerization domain (^429^PKRRR^433^). Furthermore, although Pin1 is not involved in the nuclear ATR isomerization (Hilton et al., 2015), other types of isomerases might be involved. Finally, it is worth noting that the nuclear ATR-S428A protein shifting to *trans*-ATR is likely caused by the same mechanism that maintains wild-type ATR in the *trans*-isoform in the nucleus, and the process is probably phosphorylation-independent. ATR-S428A was generated only to make ATR phosphorylation-deficient at S428 so that Pin1 whose activity depends on the phosphorylation cannot convert *cis*-ATR to *trans*-ATR in the cytoplasm. While determination of the mechanisms is of great interest, it is out of the scope of the current study and subjected to future investigation.

In particular, our results support a structural alteration around the BH3 domain of ATR (Figure 2B, Supplementary Table S1) between *trans* and *cis*/WT ATR, making the BH3 domain in *cis*/WT ATR accessible to biotin modification and, thus, likely available for protein–protein interactions including ATR–tBid interaction (Hilton et al., 2015). In addition, conformational changes may also occur involving the N-HEAT and FAT domains.

Earlier studies showed that localization of ATR to mitochondria may occur through the binding of ATR-H to mitochondria-bound tBid *via* ATR’s BH3-like domain. The binding of ATR-H to tBid leads to tBid sequestration, serving a role in preventing Bax/Bak activation. The interaction of the tBid’s BH3 domain with antiapoptotic Bcl-XL protein reportedly involves the BH3, BH1, and BH2 domains from Bcl-XL by a BH3-in-groove mechanism (Chou et al., 1999; Czabotar et al., 2013). In this study, employing the protein footprinting approach, we have mapped the surface topology of *cis/trans* ATR isomers and identified two biotin-modifiable surface lysine residues around the BH3 domain at position Lys-459 and Lys-469 in the *cis*-ATR isoform (ATR-H), but the same residues are inaccessible in *trans*-ATR (Figures 2C, Figure 3). This suggests that in *cis*-ATR, the BH3 domain undergoes a conformational change which favors ATR–tBid interaction at mitochondria, which is further supported by coimmunoprecipitation and proximity ligation assays (Figures 3D,E).

Our mass footprinting data also reveal possible conformational changes involving N-terminal HEAT repeats (N-HEAT) and FAT domains between *trans-* and *cis*-ATR isoforms (Figures 2B,C). Interestingly, the middle HEAT domains (M-HEAT) in both *cis*- and *trans*-ATR remain intact after tryptic digestion (Figure 2B) and are not, or at least not significantly, involved in the conformational change as there were no differential biotin modifications between the M-HEAT domains of these two isomers. In contrast, the N-HEAT and FAT domains of *trans*-ATR are much more surface accessible than the same domains of *cis*-ATR (Figure 2C). Given that the M-HEAT domain remains the same for both isomers, a possible scenario is that in *cis*-ATR, parts of the N-HEAT and FAT domains undergo conformation changes by being folded toward each other in close contact around the M-HEAT domain, so that parts of the original accessible surface residues in both N-HEAT and FAT domains in *trans*-ATR now become inaccessible (biotin-unmodifiable). The conformational changes also may expose the hidden BH3 domain.

ATR checkpoint function is dependent on ATR–ATRIP hetero-dimeric complex formation. Through mass footprinting and coimmunoprecipitation analyses, we found that even for mitochondrial function, *cis*-ATR maintains a dimeric state *via* direct homo-dimerization of its C-terminal domains, without the involvement of ATRIP. Now one question may arise whether conformation change of *cis*-ATR diminished ATR–ATRIP complex formation in the cytoplasm. Since there is no or very little ATRIP present in the cytoplasm, it is not possible to experimentally ascertain whether *cis*-ATR can form a *cis*-ATR–ATRIP complex in the cytoplasm.

Mass footprinting data identified two interesting lysine modifications at residue 1994 which is near the ATR auto-catalytic site T1989 and another within the ATR kinase domain at residue 2,413 in *trans*-ATR but not in *ci*s-ATR (Figure 2C). This suggests a differential conformation between *cis*- and *trans*-ATR around the FAT and kinase domains, which may affect the kinase activity of ATR. As nuclear ATR always remains in the *trans*-isoform and is ATR kinase active, it is possible that *cis*-ATR may not be kinase-active due to these structural alterations in addition to the absence of cytoplasmic ATRIP. Interestingly, we observed identical kinase activity and autophosphorylation at T1989 for all nuclear ATR WT and mutant proteins (Figures 5A,B). This is consistent with the fact that all nuclear ATR proteins are *trans*-ATR regardless of their state in the cytoplasm, which is also true for the ATR-S428A mutant. This may be due to the nuclear interaction with ATRIP and other ATR activator proteins, which force ATR-S428A to undergo a conformational conversion to *trans*-ATR.

Our findings provide a useful insight into how ATR regulates the mitochondrial cell death pathway and the nuclear DNA damage-signaling pathway by switching ATR between its *cis* and *trans* isomerization states.

## Data Availability

The mass spectrometry-based proteomics datasets presented in this study can be found in online repositories (Deutsch et al., 2019; Perez-Riverol et al., 2022). The names of the repository/repositories and accession number(s) can be found at: available *via* ProteomeXchange with identifier PXD031385 and 10.6019/PXD031385.
