# Supplementary material for: Prolyl Isomerization-Mediated Conformational Changes Define ATR Subcellular Compartment-Specific Functions
Source: Front Cell Dev Biol. 2022 Jun 3;10:826576. doi: 10.3389/fcell.2022.826576 (PMC9204103; doi:10.3389/fcell.2022.826576)
Supplement: Supplementary file 1 [file DataSheet1.PDF]

Supplementary Table S1. Biotin-modified sites on ATR-WT, -S428A and -P429A. The table includes the residues with an A-score value of 50 or above and a localization probability of 1. Biotin-modified lysine residues are marked by '+' and unmodified are marked by '-'.

Site	A-score	WT (cis)	S428A (cis)	P429A (trans)
**K32**	343.38	+	+	+
**K121**	73.06	+	+	+
**K297**	70.75	+	+	+
**K442**	299.75	+	+	+
**K446**	74.96	+	+	+
**K452**	99.48	+	+	+
**K459**	67.08	+	+	-
**K469**	91.31	+	+	-
**K518**	50.17	+	+	+
**K523**	107.96	+	+	+
**K545**	82.91	+	+	+
**K565**	70.88	+	+	+
**K690**	70.98	+	+	+
**K695**	122.33	+	+	+
**K704**	65.81	+	+	+
**K746**	64.66	+	+	+
**K759**	124.77	+	+	+
**K764**	70.87	+	+	+
**K818**	235.2	-	-	+
**K918**	161.94	+	+	+
**K1005**	143.16	-	-	+
**K1019**	108.74	+	+	+
**K1057**	50.51	-	-	+
**K1165**	148.53	+	+	+
**K1313**	118.81	+	+	+
**K1317**	124.16	+	+	+
**K1467**	74.69	+	+	+
**K1600**	167.75	+	+	+
**K1603**	57.42	+	+	+
**K1616**	236.24	+	+	+
**K1703**	115.38	-	-	+
**K1824**	288.53	+	+	+
**K1994**	215.84	-	-	+
**K2060**	73.89	+	+	+
**K2106**	183.08	+	+	+
**K2121**	167.75	+	+	+
**K2208**	90.46	-	-	+
**K2205**	55.92	+	+	+
**K2315**	178.66	+	+	+
**K2327**	72.24	+	+	+
**K2404**	189.00	+	+	+
**K2413**	139.37	-	-	+
**K2420**	50.64	+	+	+
**K2567**	56.44	+	+	+

**K2570**	54.16	+	+	+
**K2574**	54.18	+	+	+
**K2589**	54.74	+	+	+
**K2604**	212.37	+	+	+

Supplemental Table S2. Calculated total free energies and components of wild type and mutant ATR peptides. (WT: NLSSNSDGIS$_{428}$P$_{429}$KRRRLSSSL; S428A: NLSSNSDGIA$_{428}$P$_{429}$KRRRLSSSL; P429A: NLSSNSDGIS$_{428}$A$_{429}$KRRRLSSSL).
* All energies are reported in kcal/mol, P-values are from two-sided student t-test.

Energy Terms*	WT-Cis		WT-Trans		P429A-Cis		P429A-Trans		S428A-Cis		S428A-Trans	
	**Mean**	**S.D.**	**Mean**	**S.D.**	**Mean**	**S.D.**	**Mean**	**S.D.**	**Mean**	**S.D.**	**Mean**	**S.D.**
Bond	63.33	7.39	63.29	6.29	62.46	7.54	62.18	6.79	62.98	7.44	63.45	7.42
Angle	161.57	11.10	157.82	9.85	152.70	9.59	150.04	9.89	157.10	10.71	160.41	9.76
Dihed	260.58	6.82	259.24	7.17	252.07	6.90	249.63	7.45	262.98	6.82	256.14	8.12
VDW	-97.07	9.68	-90.12	7.41	-89.64	13.83	-89.73	8.17	-105.64	8.90	-92.99	14.71
ELE	-922.34	40.01	-875.39	43.49	-886.11	54.29	-883.76	41.21	-900.55	46.40	-886.41	38.02
1-4 VDW	66.82	4.04	66.62	3.68	65.09	3.81	66.54	3.90	66.49	3.31	66.42	3.64
1-4 EEL	131.70	20.58	142.86	21.41	159.14	21.37	154.66	22.64	165.95	20.76	155.56	19.15
G-Gas	-335.41	34.73	-275.69	40.48	-284.29	59.27	-290.43	36.72	-290.68	39.01	-277.41	35.82
G-Solv	-420.37	26.86	-476.51	31.59	-479.57	50.77	-480.45	28.78	-462.46	33.85	-464.60	28.23
**Total**	**-755.78**	**15.78**	**-752.20**	**15.83**	**-763.85**	**16.76**	**-770.88**	**15.90**	**-753.14**	**14.97**	**-742.01**	**17.13**
**P-value**	**0.024**				**2.22e-5**				**2.14e-11**			

Supplementary Figure 1

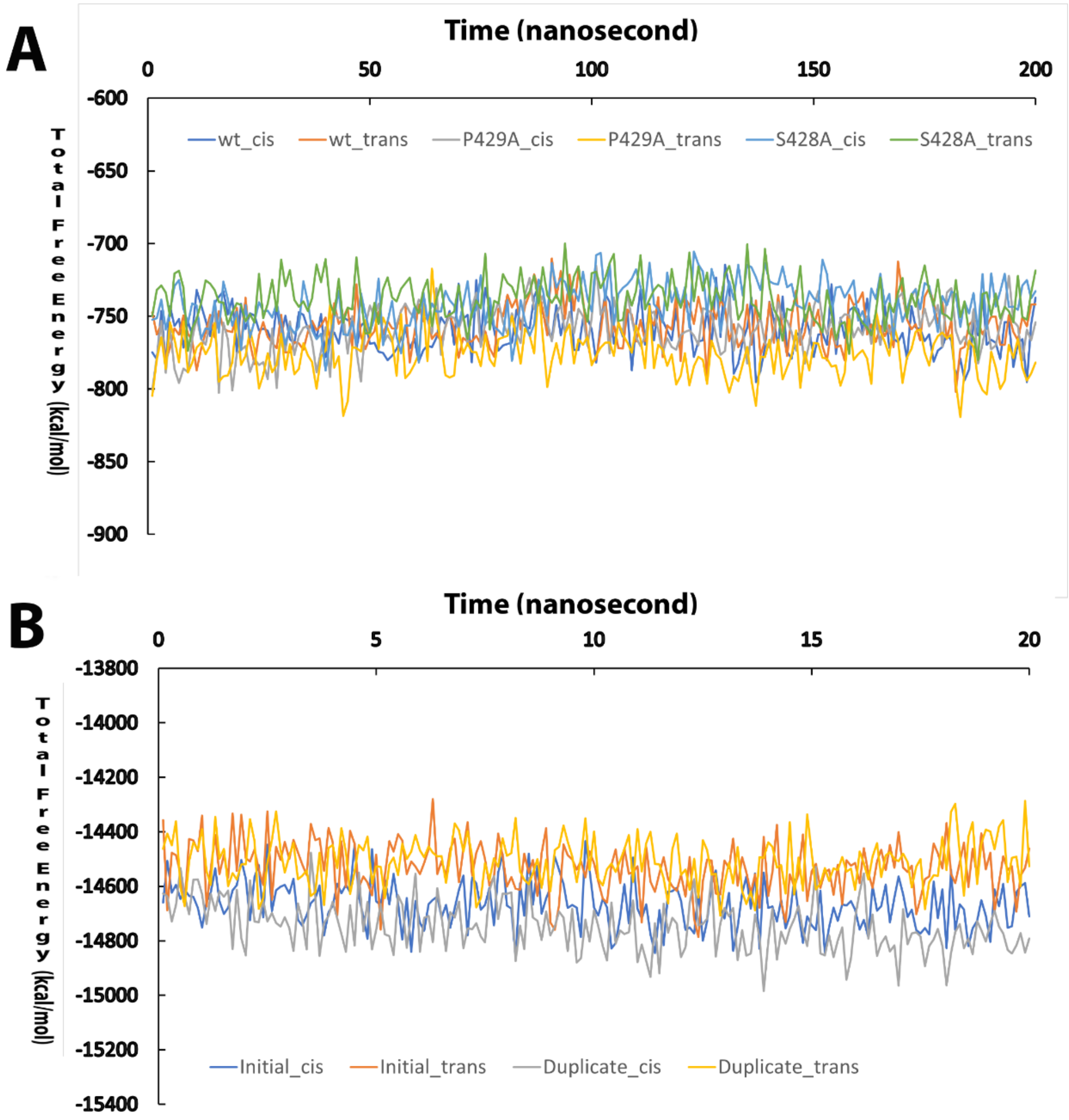

Figure S1. A) Total free energies extracted from MM-GBSA calculations along simulation time for 20aa peptides. B) Total free energies extracted from MM-GBSA calculations along simulation time for N-terminal cis- and trans-ATR 770 residues. Data from both initial and duplicated runs are shown.
